# Analog to Digital Diagnosis and Planning in Orthognathic Surgery: A Narrative Review

**DOI:** 10.7759/cureus.80858

**Published:** 2025-03-19

**Authors:** Sujeeth K Shetty, Rishabh Kasrija

**Affiliations:** 1 Department of Oral and Maxillofacial Surgery, Jagadguru Sri Shivarathreeshwara (JSS) Dental College and Hospital, Mysuru, IND

**Keywords:** diagnosis, digital planning, maxillofacial imaging, orthognathic surgery, treatment plan

## Abstract

Orthognathic surgery is a transformative procedure for correcting skeletal and dental discrepancies and enhancing facial aesthetics, functionality, and quality of life. Patients with dentofacial deformities often experience mastication, speech, and respiration challenges. Advances in diagnostic tools, treatment planning, and surgical methodologies have significantly improved surgical precision and predictability. Accurate diagnosis relies on clinical assessments, cephalometric analysis, and advanced imaging techniques such as cone beam computed tomography and three-dimensional facial scanning. Traditional surgical planning using two-dimensional cephalometry and plaster models has been largely replaced by virtual surgical planning, which enables precise simulations, computer-aided design/computer-aided manufacturing-assisted surgical splint fabrication, and improved postoperative stability. Achieving a balance between function and aesthetics is crucial. Contemporary approaches emphasize both skeletal correction and soft tissue response, with airway management being a key consideration, particularly in cases of obstructive sleep apnea. The treatment pathway includes presurgical orthodontics, maxillary or mandibular repositioning, and postoperative rehabilitation. Artificial intelligence and augmented reality-assisted surgery drive future innovations to enhance surgical accuracy and treatment customization. Despite these advancements, challenges such as treatment duration, patient compliance, psychological readiness, and costs remain. Predicting soft tissue behavior and ensuring long-term stability require further research. This review aims to provide a comprehensive overview of advancements in orthognathic surgery, from diagnostic innovations to modern treatment planning and emerging technologies, highlighting their impact on surgical precision, efficiency, and patient outcomes. With continuous technological progress and interdisciplinary collaboration, orthognathic surgery is moving toward greater precision, efficiency, and improved patient outcomes.

## Introduction and background

Orthognathic surgery constitutes a revolutionary treatment for correcting skeletal and dental discrepancies, markedly improving facial aesthetics, functionality, and the overall quality of life. Individuals afflicted with dentofacial deformities frequently encounter challenges in mastication, phonation, and respiration, in addition to psychological and social impediments [1]. Progress in orthognathic surgery has been propelled by innovations in diagnostic capabilities, meticulous treatment planning, and advanced surgical methodologies, facilitating outcomes that are both more precise and predictable [2]. By amalgamating state-of-the-art imaging techniques, virtual surgical planning (VSP), and computer-assisted technologies, the discipline has transitioned toward a patient-centric, precision-oriented paradigm that guarantees functional efficacy and aesthetic equilibrium [3].

Successful orthognathic surgery begins with an accurate diagnosis that relies on a combination of detailed clinical evaluations, cephalometric analysis, dental impressions, and advanced imaging techniques such as cone beam computed tomography (CBCT) and three-dimensional (3D) facial scanning [4]. These diagnostic tools provide a comprehensive understanding of craniofacial structures, allowing surgeons to develop individualized treatment plans. Traditionally, two-dimensional (2D) cephalometric-guided surgical planning has been used. However, the advent of 3D imaging and VSP has revolutionized the accuracy and efficiency of treatment [3]. VSP enables surgeons to perform detailed simulations of jaw movements, predict postsurgical soft tissue adaptations, and fabricate custom surgical splints using computer-aided design and manufacturing (CAD/CAM) technology. These advancements have improved surgical precision, minimized intraoperative uncertainties, reduced operative time, and enhanced postoperative stability [5].

A crucial aspect of orthognathic surgery is balancing function and aesthetics. Patients often seek treatment not only to improve masticatory function and alleviate symptoms related to malocclusion but also to enhance facial aesthetics and overall appearance. With the paradigm shift from skeletal to soft tissues, contemporary techniques attempt to strike a balance between them. Furthermore, airway management has become essential in surgical planning, particularly in obstructive sleep apnea cases, where maxillary advancement is often preferred over mandibular setback to maintain upper airway patency and prevent long-term respiratory complications [6].

The roadmap for orthognathic surgery follows a structured pathway, beginning with pre-surgical orthodontic treatment to align the teeth within each jaw before surgical repositioning. This phase typically lasts for several months and ensures optimal post-surgical occlusion. The surgical phase involves repositioning the maxilla, mandible, or both, depending on the nature of the skeletal discrepancy. In some cases, adjunctive cosmetic procedures such as genioplasty may be performed to refine facial contours [7]. Postoperative care is equally critical, encompassing pain management, dietary modifications, and physiotherapy to aid recovery and adaptation to the new jaw position. Long-term stability is a key objective, with post-surgical orthodontic refinement ensuring that the occlusion remains stable while soft tissues adapt to the underlying skeletal changes [8].

With continuous advancements in digital technology, artificial intelligence (AI), and biomechanical simulations, the future of orthognathic surgery is shifting toward greater precision and patient-specific customization. Innovations such as augmented reality-assisted surgery (ARAS) and robotic-assisted interventions (RAI) hold promise for further enhancing surgical accuracy and minimizing complications [9]. As the field progresses, interdisciplinary collaboration between orthodontists, maxillofacial surgeons, and prosthodontists will remain vital for optimizing patient outcomes.

This review outlines the roadmap for orthognathic surgery from initial diagnosis to precision treatment, highlighting the pivotal role of modern technology in achieving functional, aesthetic, and long-term stability in patients undergoing jaw correction procedures.

## Review

Search criteria and methodology

To ensure a comprehensive review, an extensive literature search was conducted using four databases: PubMed, Google Scholar, Scopus, and Cochrane Library. The search terms included “orthognathic surgery”, “virtual surgical planning”, “3D imaging in maxillofacial surgery”, “CAD/CAM in orthognathic surgery”, “psychological impact of jaw surgery”, “airway considerations in orthognathic surgery”, “surgical splints”, “model analysis in orthognathic surgery”, and “cephalometrics in orthognathic surgery”. Studies and review articles from 2000 were included, mainly randomized control trials and systematic reviews. Articles that focused solely on orthodontic treatment without surgical intervention were excluded.

Patient selection and psychological assessment for orthognathic surgery

**Figure 1:**
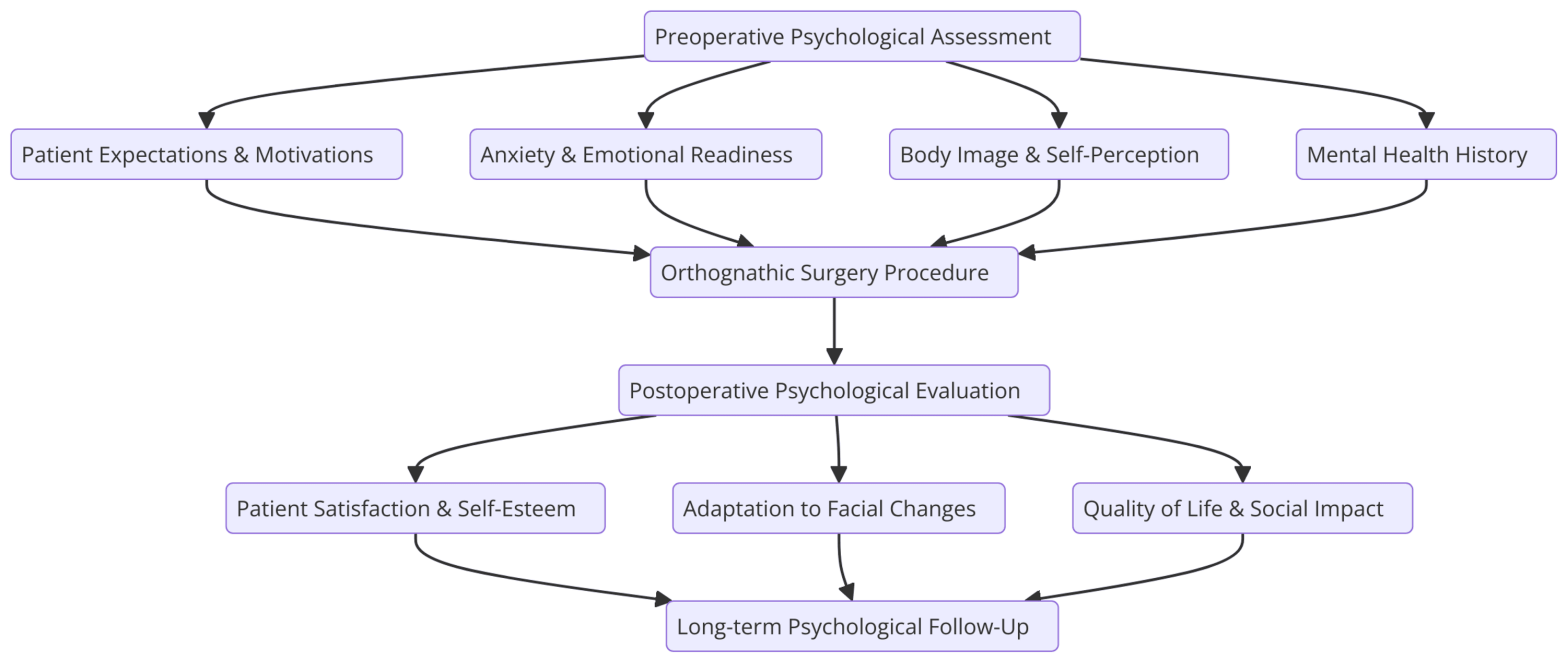
Psychological assessment in orthognathic surgery The image is the property of the authors and is relevant to the content of the study.

Patient selection for orthognathic surgery requires a comprehensive assessment of both functional needs and psychological readiness. While advancements in surgical technology have expanded the treatment options, patient motivation remains a key determinant of success. Candidates typically present with severe malocclusion, temporomandibular dysfunction, or significant facial asymmetry, which impair oral function and aesthetics. Beyond physical considerations, psychological evaluation is crucial, as patients with a positive body image and realistic expectations tend to achieve better outcomes (Figure 1).

Factors such as long-standing dissatisfaction, external pressure, and anxiety must be carefully assessed. Effective communication and counseling help prepare patients for the surgery's emotional and physical impact [10].

Detailed clinical examination of the patient

A detailed clinical examination is crucial for accurate diagnosis and optimal planning in orthognathic surgery. It begins with a thorough medical and dental history, assessment of systemic conditions, previous treatment, and patient concerns. Extraoral evaluation focuses on facial symmetry, skeletal proportions, and soft tissue balance, analyzing the facial thirds, lip posture, and chin position for aesthetic considerations. Intraoral assessment included occlusion analysis, overjet, overbite, midline discrepancies, and dental compensation. Periodontal health, caries, and temporomandibular joint (TMJ) function were examined because TMJ disorders may influence surgical decisions. Functional evaluation includes mastication, speech, swallowing, and airway patency, with special attention paid to the risk of obstructive sleep apnea [11]. A psychological assessment ensures patients have realistic expectations and emotional readiness for surgery [10]. A multidisciplinary team, including orthodontists, maxillofacial surgeons, and psychologists, improved treatment predictability and patient satisfaction.

Traditional surgical planning

Before the digital advancements, traditional surgical planning (TSP) was performed using clinical photographs, plaster models, and 2D radiographs, including cephalometric analysis, to diagnose skeletal and dental discrepancies. Dental casts allow occlusal evaluation, whereas articulators simulate jaw movement. Cephalometric radiographs, introduced in the 1930s, provide standardized skeletal and dental measurements for treatment planning. However, these methods have limitations, including the inability to capture soft-tissue dynamics and complex asymmetries. TSP is a cost-effective approach; however, it has largely been supplemented by 3D imaging and VSP, which offer greater precision in orthognathic surgery by improving diagnostic accuracy, treatment predictability, and patient outcomes [12].

Initially, a traditional 2D approach was used to fabricate surgical splints, in which mock surgeries were performed using a semi-adjustable articulator. However, interocclusal splints provide limited control over the maxillary 3D position relative to the basal skull, with reported misalignments of up to 5 mm [13]. Independent maxillary positioning is challenging and requires intermediate splints, which complicate counterclockwise rotation and condylar positioning within the TMJ [14]. Various methods address these traditional orthognathic planning limitations, including the double-splint technique, face-o-meter, and nasal pin, although they mainly assess medial frontal measurements, excluding posterior maxillary landmarks and inclinations [3].

To address these drawbacks, 3D-COSMOS, a craniofacial halo apparatus that facilitates the transfer of 3D planning and demonstrates encouraging outcomes, was developed [15]. Subsequently, logical progression involved the incorporation of computer-assisted methodologies, such as navigational systems and CAD/CAM splints, into the procedural framework. Zinser et al. [16] evaluated the accuracy of surgical splints fabricated using the traditional, CAD/CAM, and navigation methods. The highest precision was observed for CAD/CAM, followed by navigation, and the lowest was observed for splints fabricated using traditional methods. They also suggested that there is now a paradigm shift in the diagnosis and treatment planning for orthognathic surgery from TSP to VSP for better precision.

Virtual surgical planning

**Figure 2:**
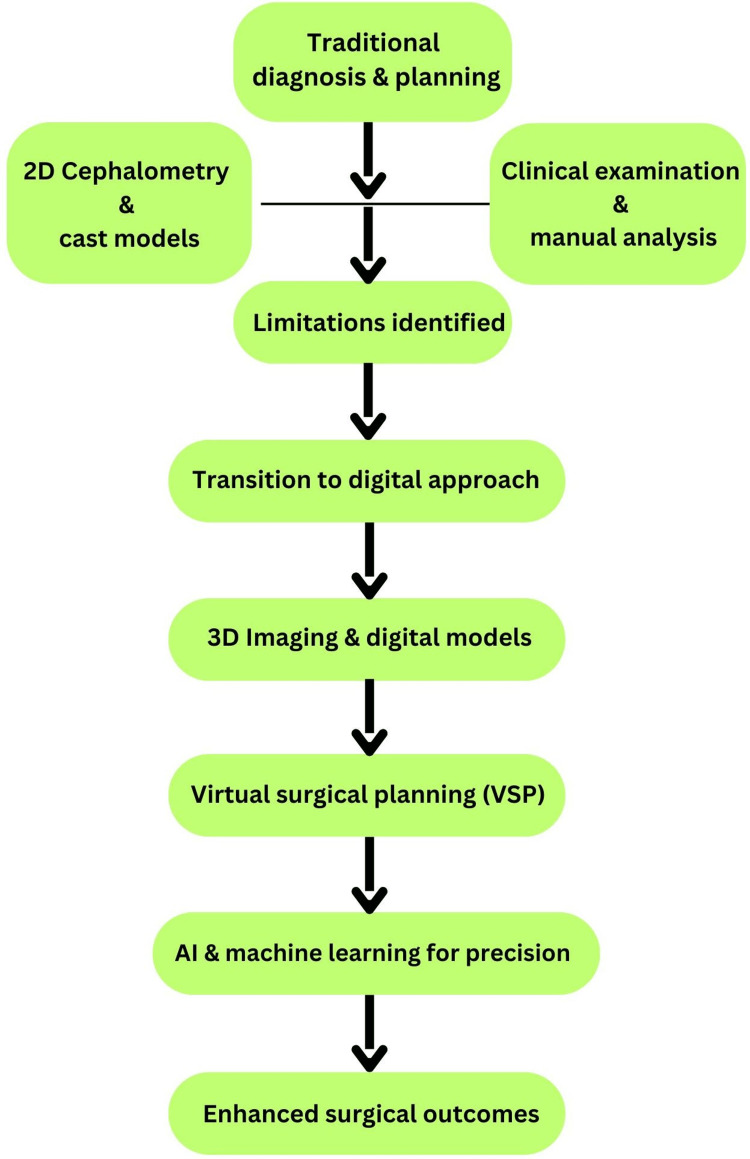
Changing parameters of traditional orthognathic surgery to VSP 2D: two dimensional, 3D: three dimensional, VSP: virtual surgical planning, AI: artificial intelligence The image is the property of the authors and is relevant to the content of the study.

Although VSP has been posited as a more advantageous alternative to TSP, scholars have questioned whether the accuracy of the VSP methodology surpasses that of TSP. However, TSP has been recognized as superior from a cost-efficiency perspective. Nevertheless, the cost-benefits associated with TSP were evident after the preliminary fixed-cost outlay required for VSP. In their comprehensive systematic review and meta-analysis, Chen et al. [17] established that VSP and TSP exhibited comparable predictive accuracies in the sagittal plane (Figure 2).

The study delineated TSP as a systematic procedure necessary for completing preoperative planning, encompassing radiographic evaluations, 2D analyses, model surgical practices, and the fabrication of surgical splints. Conversely, VSP was characterized as the methodological instrument for forecasting intricate surgical movements in a 3D space, including radiographic assessments, clinical photographic documentation, production and scanning of plaster models, virtual analytical evaluations, and 3D printing of surgical splints [18,19]. According to Park et al. [18], the time invested in VSP was significantly lower than in TSP. This method is particularly applicable in bimaxillary surgical cases [19].

Hanafy et al. [20] reported that VSP presents significant advantages over TSP, particularly for trainees and novice surgeons. This distinction arises from its ability to accurately discern bone and root morphology, in contrast to the TSP. These results underscore that both TSP and VSP are instrumental in providing the requisite guidance for the successful execution of surgical procedures.

To create an accurate digital model of a patient's anatomy, VSP begins with high-resolution 3D imaging, such as CBCT or facial scanning. Using specialized software, surgeons can analyze skeletal discrepancies and simulate surgical correction. This allows precise bone repositioning and occlusal adjustment. Custom surgical guides were used to ensure intraoperative accuracy. VSP also enhances patient communication by visualizing expected outcomes and improving informed decision-making. During surgery, these digital plans guide precise execution, reduce errors, and improve the results. Overall, VSP has revolutionized orthognathic surgery, offering enhanced precision, efficiency, and predictability compared with traditional planning methods [21,22].

Role of artificial intelligence and augmented reality

The future of orthognathic surgery lies in AI and ARAS. AI can help to refine treatment algorithms and predict surgical outcomes with greater accuracy. AR can enhance intraoperative visualization, aid real-time surgical navigation, and minimize errors [9]. However, their widespread implementation requires further validation and regulatory approval. According to Du et al. [23], the binary relevance extreme gradient boosting model has more than 90% diagnostic accuracy for six dentofacial deformities. Czako et al. [24] found that AI in orthognathic surgery primarily enhances diagnostics, treatment planning, and postoperative evaluation with increased integration into maxillofacial and robotic surgery for comprehensive surgical assessment and improved outcomes.

According to Miragall et al. [25], AI has great potential in the field of orthognathic surgery, where it can support surgeons in orthognathic surgery, aiding preoperative planning, virtual simulations, and intraoperative decisions to improve accuracy, reduce complications, and predict bleeding risks. Almarhoumi [26] conducted a scoping review to assess the accuracy of AI in predicting postsurgical facial changes, and seven studies met the inclusion criteria. It was concluded that AI demonstrated significant potential for accurately predicting postsurgical facial changes after orthognathic surgery. Polley and Figueroa [27] described an occlusion-based orthognathic positioning system, which transfers VSP to the surgical site to appropriately reposition osteotomized segments. It comprises a detachable guide that is fixed to an occlusal splint. It includes an initial drilling guide for stable surgical reference marks away from the osteotomy site. Another guide helps position the skeletal segments per the predetermined VPS. This VPS is a 3D CAD/CAM technology similar to that used for stereolithic models. The VPS is equally useful in surgical planning by determining osteotomy sites and patient-specific configured miniplates, reducing the chances of postoperative occlusal disharmony. The postsurgical duration of orthodontic treatment is significantly reduced, as is patient discomfort.

Future use of artificial intelligence in biomechanical simulation

**Figure 3:**
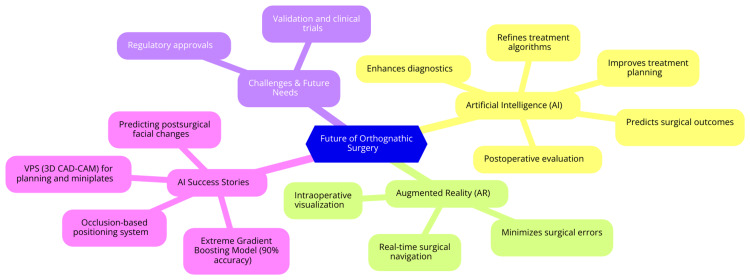
Future of orthognathic surgery with integration of AI AI: artificial intelligence, AR: augmented reality, VSP: virtual surgical planning, 3D: three dimensional, CAD/CAM: computer-aided design and manufacturing The image is the property of the authors and is relevant to the content of the study.

Advances in biomechanics are expected to improve the predictability of treatment. AI-driven biomechanical simulations may allow more personalized treatment strategies considering patient-specific anatomical and physiological variations [28]. Future studies should focus on refining simulation models to enhance surgical precision (Figure 3).

Limitations in treatment planning

Treatment planning for orthognathic surgery is a complex process influenced by various limitations that must be carefully considered. One of the primary challenges is individual variation, as each patient’s facial anatomy, skeletal structure, and dental occlusion differ and require personalized treatment approaches. Factors such as bone density, muscle attachment, and soft tissue response can affect surgical outcomes and may necessitate modifications during the procedure. Accurate diagnostic records are essential for precise planning. Errors in these records can lead to inaccuracies, potentially compromising the surgical outcomes. Another challenge is predicting soft tissue behavior because factors such as age, muscle tone, and scarring influence soft tissue adaptation to skeletal repositioning. The stability of the surgical results is also a concern, as relapse may occur due to surgical complexity, healing patterns, or patient compliance. Furthermore, certain surgical techniques may have inherent limitations that restrict the extent of the achievable movements. Effective interdisciplinary coordination among oral and maxillofacial surgeons, orthodontists, and other specialists is crucial to achieving successful outcomes. Lastly, patient compliance with pre- and postoperative care and realistic expectations play a significant role in determining the success of orthognathic surgery. According to Klein et al. [29], inadequate treatment planning can lead to surgical complications and inadequate results.

Challenges

Long Treatment Duration and Patient Compliance

Orthognathic treatment typically spans several years, beginning with preoperative orthodontics followed by surgery and postoperative orthodontic refinement. Patient compliance is crucial but is often a limiting factor. Orthodontic treatment delays can extend the timelines and reduce patient satisfaction and adherence to treatment plans. AlKharafi et al. [30] reported that most patients who underwent orthognathic surgery felt they should provide more information about the surgical discomfort and associated risks.

Psychological and Social Implications

Although orthognathic surgery significantly improves self-esteem and quality of life, preoperative psychological distress is common. Many patients experience anxiety regarding surgical risks, postoperative pain, and changes in facial appearance. Studies have highlighted the need for psychological counseling as an integral component of treatment planning [10]. Future research should explore standardized psychological screening protocols for patients with orthognathic diseases.

Cost and Accessibility

Despite the effectiveness of modern techniques, cost remains a significant barrier. Advanced digital planning tools, intraoperative navigation systems, and customized prosthetics have increased treatment expenses. Furthermore, access to high-quality orthognathic care is limited in certain regions, highlighting the need for cost-effective solutions and telemedicine integration.

## Conclusions

Orthognathic surgery has evolved significantly from traditional diagnostic approaches to precision-driven treatment planning. Advances in imaging technologies, digital diagnostics, and AI have enhanced the accuracy of skeletal and soft tissue relationship assessment, improving surgical outcomes. Integrating interdisciplinary collaboration, patient-specific planning, and emerging innovations, such as robotic-assisted surgery, continues to refine the field and ensure safer procedures with predictable results. Although challenges remain, including soft tissue response prediction and long-term stability, ongoing research and technological advancements offer promising solutions. As orthognathic surgery moves toward greater precision, a patient-centered approach combined with cutting-edge techniques is crucial for optimizing functional and aesthetic outcomes.
